# Entropy and expertise: assessing changes in pathologists' language over time using the UK Liver Pathology External Quality Assessment scheme

**DOI:** 10.1002/2056-4538.70032

**Published:** 2025-06-13

**Authors:** Jonathan P Callaghan, Katrina Z Freimane, Rachel M Brown, Alyn L Cratchley, Timothy J Kendall

**Affiliations:** ^1^ Pathology and Data Analytics University of Leeds Leeds UK; ^2^ Department of Cellular Pathology University Hospitals Birmingham NHS Foundation Trust Birmingham UK; ^3^ Department of Cellular Pathology Leeds Teaching Hospitals NHS Trust Leeds UK; ^4^ Centre for Inflammation Research Institute for Regeneration and Repair, University of Edinburgh Edinburgh UK; ^5^ Edinburgh Pathology University of Edinburgh Edinburgh UK

**Keywords:** entropy, language, EQA scheme, External Quality Assessment, liver pathology

## Abstract

External Quality Assessment (EQA) schemes are an important quality assurance tool and aim to ensure consistency among histopathologists. In this study, we use Shannon entropy as a novel metric to evaluate linguistic variability in the UK Liver Pathology EQA scheme. Analysing free‐text responses by participants over a decade, we aimed to quantify language trends in morphological assessments and clinicopathological diagnoses. Accounting for an increasing word count and when pathologists joined the scheme, our findings reveal a significant increase in entropy of morphological assessments over time, indicating growing linguistic diversity that may reflect the increasing complexity of liver pathology. Entropy of clinicopathological diagnoses over the same period did not provide clear evidence for convergent diagnostic language. High entropy corresponded to cases that elicited more diverse responses and could be considered more challenging, highlighting the utility of this method to identify potential areas for targeted education. We demonstrate entropy as a novel tool to analyse pathologist language and enhance quality assurance in the evolving pathology landscape.

## Introduction

Pathological diagnosis can be considered the ‘gold standard’ in medicine, providing crucial information to inform patient management. Consistency of diagnoses and quality assurance of the information provided in the pathological report are key objectives of the practicing pathologist, which in the United Kingdom (UK) is supported by participation in External Quality Assessment (EQA) schemes [1].

Most pathology specialities run national EQAs in the UK. The Royal College of Pathologists (RCPath) suggests that EQA schemes should support professional standards by ‘standardising and harmonising diagnostic criteria across the country’ and keep pathologists informed of developments within their specialities. Furthermore, EQAs should allow participants to reflect upon their diagnostic performance and address knowledge gaps through self‐directed learning [2]. The liver EQA scheme, coordinated by the UK Liver Pathology Group (UKLPG), aims to reflect the diversity of cases encountered in routine medical and lesional reporting, encompassing both biopsies and resections [3]. Substantial advances in our understanding of liver disease over the last 50 years have resulted in an ever‐evolving diagnostic landscape [4].

Few specialities have examined trends in EQA schemes over time. Within breast pathology EQAs [5, 6], diagnostic concordance appears to improve with time, as judged by increases in the value of the kappa statistic. However, this method for evaluation only examines whether a correct diagnosis has been made by a pathologist for each case using a binary ‘yes/no’ category assigned to each answer. This has been criticised for not reflecting individual pathologist performances in a real‐world context where diagnosis is a complex and iterative process incorporating subjective experiences and judgement [1, 7]. Furthermore, previous studies analysing EQAs [5, 6, 8] are based on grouping diagnoses as benign, *in situ*, or invasive. Although useful for examining diagnostic concordance, this approach does not capture linguistic nuance among the reporting pathologists, a phenomenon which has been described in ‘live’ reports, for example, in relation to communicating diagnostic certainty [9].

Shannon entropy, from the field of information theory, can quantify the uncertainty of a linguistic signal in text using a probability distribution. Higher entropy indicates greater variability and less repetition within the text [10]. While entropy‐based methods have not been applied to EQA schemes previously, they are important in natural language processing and recently have been used for applications such as detecting large language model hallucinations [11].

Using free‐text responses to the liver EQA circulations between 2011 and 2020, inclusively, we aimed to examine trends in language use and quantify linguistic variability using entropy. We also sought to assess whether entropy can be used as a novel metric to evaluate the key objective of EQA schemes – enhancing consistency among reporting pathologists.

## Materials and methods

The UK National Liver Histopathology EQA Scheme is open to consultant pathologists in the UK and Ireland who routinely report liver pathology in both general or subspecialist settings. All cases are submitted by scheme members and anonymised, with material shared online for educational purposes [3].

This study analysed data from 19 EQA circulations conducted between 2011 and 2020, inclusively. In 2021, the scheme transitioned to a digital only format with drop‐down options to select responses. Each circulation comprised 12 cases (glass H&E‐stained slides, with special stains and immunohistochemistry available as scanned digital images), distributed to participants who submitted free‐text responses online. Respondents in the liver EQA scheme are asked to provide both a morphological assessment, which consists of a description of the cardinal histological features and a synthesis into a pattern of injury, and a clinicopathological diagnosis, which incorporates the morphological pattern with supplied clinical information into a categorisation that fellow clinicians can understand and act upon. Consensus diagnoses that formed the annotations for each case were established at biannual meetings of the EQA committee and the UKLPG, with the ‘correct' diagnosis determined by a minimum of 80% consensus among scheme respondents [3]. In some cases, no consensus was reached.

Participating histopathologists are required to engage with at least two in every three circulations, providing longitudinal data for many participants across the study duration. Routinely collected EQA data were provided by the scheme organisers for this analysis for the purposes of examining the scheme performance. Only responses from consultant participants were included in this analysis. Each participant has a unique anonymous code, and no participant or patient identifiable information was used in this study.

In 2015 (circulation number 9), a character limit was introduced on the clinicopathological diagnosis free‐text response field. We therefore evaluated clinicopathological diagnosis data between 2011 and 2015 (circulations 1–8) and 2015 and 2020 (circulations 9–19) separately. No limit was introduced on the morphological assessment response field.

Blank entries were excluded from the analysis. Temporal trends in entropy were validated using Levenshtein distances, a string metric that measures the minimum number of single‐character edits (insertions, deletions or substitutions) needed to transform one text into another. Larger Levenshtein distances indicate greater variability in texts [12]. Entropy trends were further evaluated using linear mixed‐effect models to account for participant‐level variability. The significance of circulation number in these models was confirmed using likelihood ratio tests.

All data processing and analysis was conducted in R (V4.4.1), using the packages readxl (1.4.3), dplyr (1.1.4), ggplot2 (3.5.1), patchwork (1.3.0), tidytext (0.4.2), lme4 (1.1‐35.5), lmerTest (3.1‐3), and stringdist (0.9.12).

## Results

Across 19 circulations, 159 individual participants contributed responses, with 112 (70%) participating in at least five circulations. On average, participants responded to 90% of the circulations between the first and last circulations they participated in. After removing cases where morphological assessment or clinicopathological diagnosis fields were left blank, a total of 18,484 separate responses were included in the analysis. Cases representing 53 different diagnoses were circulated. Apart from *no consensus*, the most frequent consensus answers included *steatohepatitis*, *hepatocellular carcinoma*, *focal nodular hyperplasia*, and *autoimmune hepatitis*.

**Figure 1:**
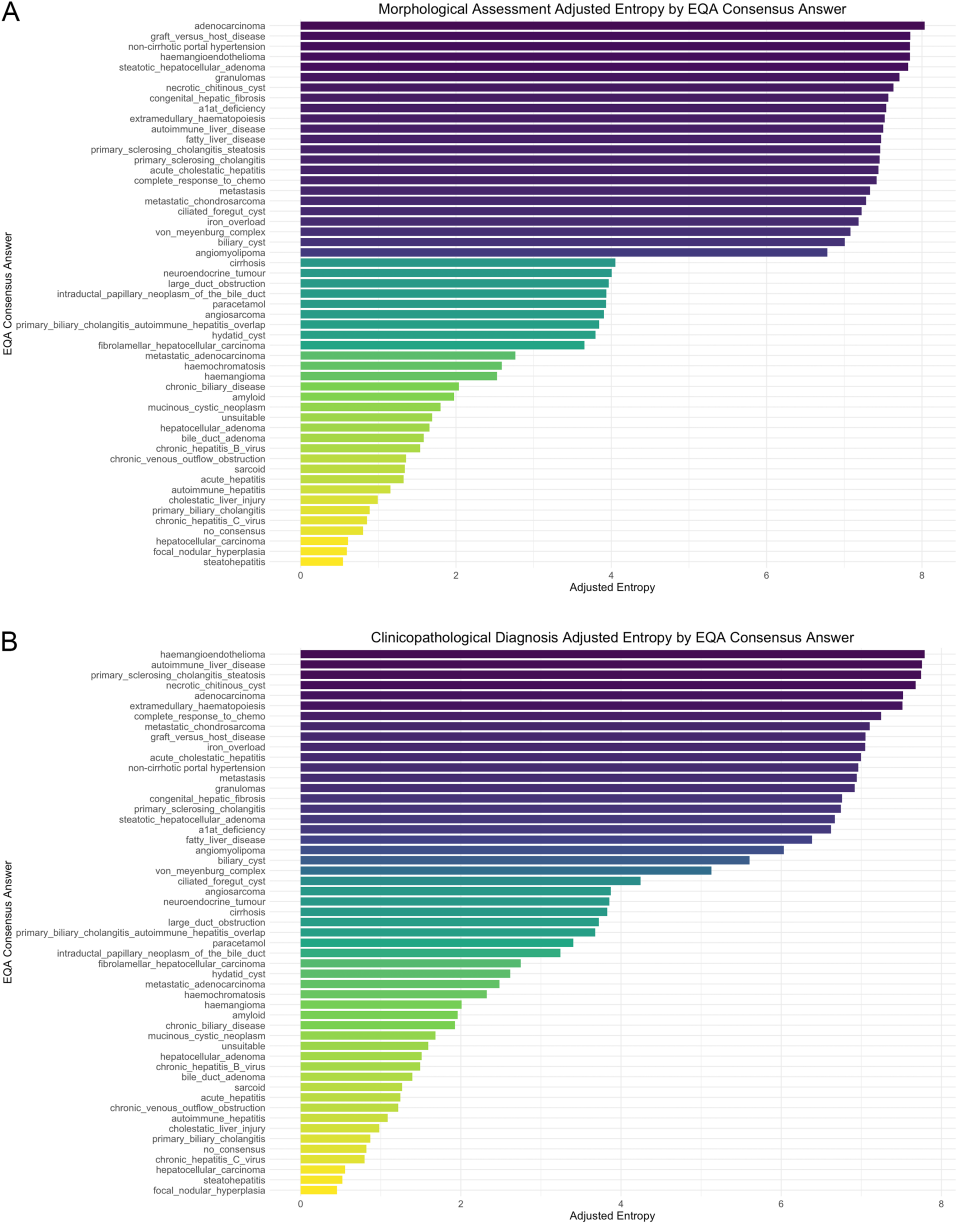
(A) Morphological assessment and (B) clinicopathological diagnosis mean entropies (bits) by EQA consensus answer, adjusted for frequency of consensus answer across all circulations (2011–2020).

Figure 1 illustrates the mean entropy for morphological assessment and clinicopathological diagnosis for each EQA consensus answer, adjusted for frequency of the diagnosis across all circulations. When diagnoses were categorised as ‘entities’ or ‘processes’ (supplementary material, File S1), the mean morphological assessment entropy was slightly higher (4.71) in the ‘processes’ group compared to the ‘entities’ group (4.51), but this difference was not statistically significant (two‐tailed *t*‐test, *p* = 0.80).

**Figure 2:**
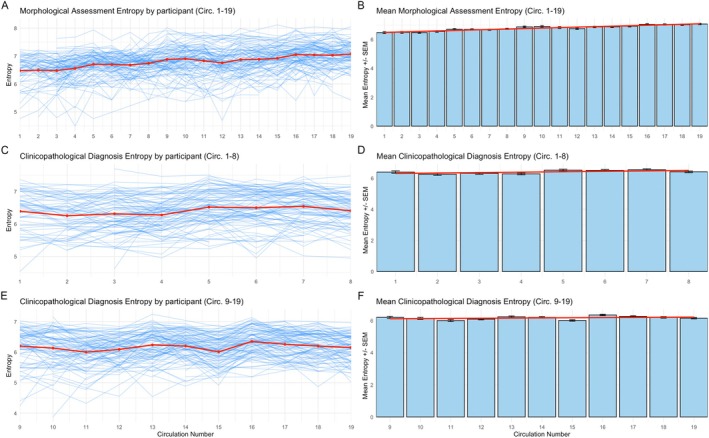
Entropy (bits) of morphological assessments and clinicopathological diagnoses across EQA circulations: (A) individual participant morphological assessment entropy across all circulations; (B) mean participant morphological assessment entropy across all circulations, with linear regression trend line; (C) individual participant clinicopathological diagnosis entropy across circulations 1–8; (D) mean participant clinicopathological diagnosis entropy across circulations 1–8, with linear regression trend line; (E) individual participant clinicopathological diagnosis entropy across circulations 9–19; (F) mean participant clinicopathological diagnosis entropy across circulations 9–19, with linear regression trend line.

**Figure 3:**
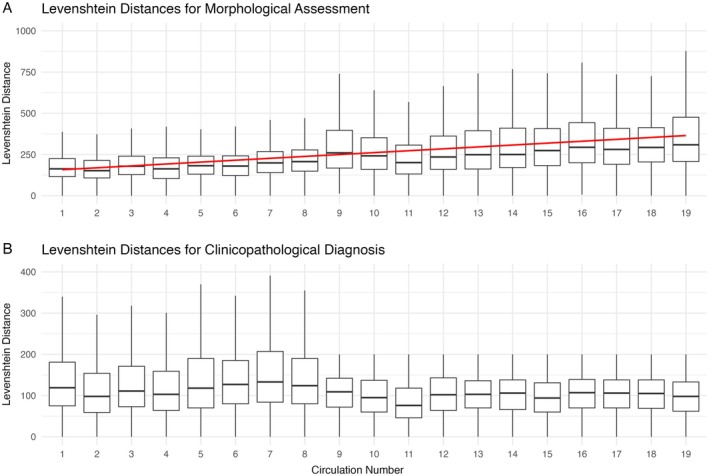
Levenshtein distances across all circulations for (A) morphological assessments and (B) clinicopathological diagnoses. Box plots display the distribution of Levenshtein distances, with the box indicating the median and the interquartile range, and whiskers extending up to 1.5 times the interquartile range. In plot (A), the red line represents the linear regression trend line for the mean Levenshtein distances of morphological assessments.

Across all circulations, entropy in morphological assessments correlated moderately with that of clinicopathological diagnoses (Pearson's *r* = 0.468, *p* < 0.001). Temporal trends in response entropy are shown in Figure 2. The trend of morphological assessment entropy increasing with time is corroborated by the trend in Levenshtein distances (Figure 3). An apparent limit on the maximum Levenshtein distances for clinicopathological diagnosis can be appreciated after circulation 9, corresponding with the introduction of the character limit.

Over time, there was a clear trend towards using more words in the morphological assessment, increasing from a mean of 21 to 47 words. Between circulations 1 and 8, the mean number of words used in the diagnosis increased from 14 to 17 but then remained level at an average of around 12 words in the subsequent circulations. Longer texts tend to be more semantically diverse, and new participants might introduce their own lexicons to the scheme; therefore, start circulation and mean word count were considered as potential confounders. Linear mixed‐effect models, accounting for participant variability, word count, and start circulation revealed that circulation number was a significant independent predictor of morphological assessment entropy (*p* < 0.001) (Table 1). This suggests a significant increase in the diversity of words used in morphological assessments as circulation number progresses. This finding was further supported by a likelihood ratio test comparing models with and without circulation number (*p* < 0.001), which confirmed that incorporating circulation number significantly improved model fit. The same significant positive trend for morphological assessment entropy was observed both before and after 2015 (circulation 9).

**Table 1:** Linear mixed‐effect models for morphological assessment and clinicopathological diagnosis entropy (bits)

Morphological assessment entropy (circulations 1–19)		
Predictor	Coefficient estimate (standard error)	*p*
Intercept	6.225 (0.0334)	<0.001*
Circulation number	0.0128 (0.0013)	<0.001*
Start circulation	−0.0047 (0.0042)	0.26
Mean word count	0.0138 (0.0005)	<0.001*

Clinicopathological diagnosis entropy (circulations 1–8)		
Predictor	Coefficient estimate (standard error)	*p*
Intercept	5.654 (0.0462)	<0.001*
Circulation number	0.0071 (0.0047)	0.132
Start circulation	−0.0071 (0.0144)	0.623
Mean word count	0.0458 (0.0017)	<0.001*

Clinicopathological diagnosis entropy (circulations 9–19)		
Predictor	Coefficient estimate (standard error)	*p*
Intercept	5.184 (0.0677)	<0.001*
Circulation number	0.0040 (0.0022)	0.068
Start circulation	−0.0082 (0.0057)	0.148
Mean word count	0.0841 (0.0021)	<0.001*

* *p* < 0.001.

Across all 19 circulations, clinicopathological diagnosis entropy demonstrated a negative trend. However, when examining the trends before and after the introduction of the character limit on the clinicopathological diagnosis response field, no significant trends were observed. As expected, mean word count was an independent significant positive predictor of entropy across all models in Table 1. Interestingly, start circulation did not predict entropy in any model. Applying these linear mixed‐effect models to ‘no consensus’ answers only, across all circulations, a significant and independent positive trend in the morphological assessment entropy was seen (*p* < 0.001). Additionally, between circulations 1 and 8, there was a small but significant positive trend in clinicopathological diagnosis entropy (*p* < 0.001); however, no such trend was observed between circulations 9 and 19.

## Discussion

This study demonstrates measurable change within the pathologists' use of language within the UK Liver Pathology EQA scheme between 2011 and 2020. To our knowledge, this is the first description of using entropy to assess and quantify variability in pathologists' language.

The pathologists' morphological assessments within the liver EQA scheme showed increasing mean word counts, entropy, and Levenshtein distances over time. The increase in entropy persisted after accounting for participant level variability, mean word count and start circulation, indicating a significant and independent rise in linguistic variability between the first and the last circulation analysed. This could reflect the growing complexity in liver pathology, something acknowledged by RCPath [13]. Interestingly, consensus diagnoses with high diagnostic entropy often corresponded to a high entropy in the descriptions used for morphological assessment. Two diagnoses with the highest diagnostic/morphological entropy were haemangioendothelioma and graft‐versus‐host disease. The former presents a diagnostic challenge due to its rarity and is misidentified in 60–80% of cases [14], while the latter may be histologically indistinguishable from drug‐induced liver injury [15]. Identifying cases that elicit more diverse responses from liver pathologists could help highlight challenging conditions that require increased educational input. However, caution is needed – for example, the high entropy for a ‘metastasis’ diagnosis is likely attributable to the variety of metastatic origins rather than inconsistent reporting. Similarly, low entropy may not always indicate diagnostic proficiency but could instead reflect conformity to a consensus, a common criticism of EQA scheme validity [7]. Nevertheless, these findings underscore the value of free‐text responses in capturing nuanced information, which may be lost with the recent switch to dropdown answers in this EQA scheme. Consideration should be given to a hybrid approach incorporating both free‐text and standardised formats to balance consistency with the need for reflective practice, particularly for challenging cases.

Notably, when examined before and after the introduction of the character limit in circulation 9, no significant trends in clinicopathological diagnosis mean word count, entropy, or Levenshtein distances were observed. Given the introduction of guidelines such as the RCPath liver tissue pathways [13, 16], an increase in diagnostic concordance might have been expected, mirroring trends seen in other pathology specialties [5, 6]. If pathologists had adopted increasingly similar language over time, a corresponding decrease in diagnostic entropy with successive circulations might be expected; however, this was not observed. This may have been influenced by the increasing variability seen in morphological assessments. Furthermore, the relationship between diagnostic entropy and diagnostic concordance, or other measures of scheme effectiveness, remains uncertain. One EQA scheme previously showed a plateau in reporting concordance a few years after implementation [5]. It is possible that a similar plateau was not captured in our study due to the timeframe analysed or constraints imposed by the character limit on the clinicopathological diagnosis field.

The evidence for a quantifiable change in pathologists' language over time presents important implications for artificial intelligence (AI) models trained on historic histopathological reports. In their survey, McGenity *et al* [17] describe the optimistic yet wary views of liver pathologists towards use of AI in their specialty. AI tools often rely on existing data for training, which poses challenges for their generalisability and performance monitoring [18, 19]. The effectiveness of these tools, such as large language models, is closely tied to the quality and consistency of the datasets used for their development [19]. Given the longitudinal changes demonstrated here, it is crucial to consider whether AI tools trained on static datasets may overlook evolving trends in pathologists' language over time.

This study is limited by its focus on a single EQA scheme and findings may not be generalisable to other specialties. Further work examining whether similar entropy patterns occur in clinical reporting, other pathology specialties, or even other disciplines such as radiology could be insightful.

## Conclusion

This study is the first to demonstrate, through analysing entropy, that pathologists' language in morphological descriptions within the UK National Liver Pathology EQA scheme has become increasingly varied over time. In contrast, linguistic variability in diagnoses did not show any clear trends; however, this might be attributable to introduction of a character limit. Our findings highlight entropy as a novel tool to analyse pathologists' use of language which might be useful, for example, to identify challenging cases that may benefit from targeted educational interventions. In the digital pathology era, pathologists' language changing over time has important implications for quality assurance and ultimately patient care.

## Author contributions statement

JPC: study conception, data analysis and writing of manuscript. KZF: writing of manuscript. RMB and ALC: data acquisition and critical revision of manuscript. TJK: study conception, data acquisition and critical revision of manuscript. All authors made substantial contributions to the analysis and interpretation of data and approved the final version of the manuscript for submission.

## Supporting information


**File S1.** Entities and processes

## Data Availability

Routinely collected EQA data were provided by the scheme organisers for this analysis for the purposes of examining the scheme performance. These data are not publicly available given scheme participants have not been consented for the sharing of individual level responses.
