# Supplementary material for: Entropy and expertise: assessing changes in pathologists' language over time using the UK Liver Pathology External Quality Assessment scheme
Source: J Pathol Clin Res. 2025 Jun 13;11(4):e70032. doi: 10.1002/2056-4538.70032 (PMC12163543; doi:10.1002/2056-4538.70032)
Supplement: Supplementary file 1 — File S1. Entities and processes [file CJP2-11-e70032-s001.pdf]

Entropy and expertise: assessing changes in pathologists' language over time using the UK Liver Pathology external quality assessment scheme

JP Callaghan *et al.* *J Pathol Clin Res* https://doi.org/10.1002/2056-4538.70032

Supplementary File S1

Entities and processes

Entities = "adenocarcinoma", "bile_duct_adenoma", "steatotic_hepatocellular_adenoma", "metastatic_adenocarcinoma", "hepatocellular_carcinoma", "hepatocellular_adenoma", "angiomyolipoma", "mucinous_cystic_neoplasm", "biliary_cyst", "fibrolamellar_hepatocellular_carcinoma", "haemangioendothelioma", "focal_nodular_hyperplasia", "amyloid", "necrotic_chitinous_cyst", "metastatic_chondrosarcoma", "intraductal_papillary_neoplasm_of_the_bile_duct", "sarcoid", "neuroendocrine_tumour", "ciliated_foregut_cyst", "von_meyenburg_complex", "angiosarcoma", "metastasis", "hydatid_cyst", "haemangioma"

Processes = "chronic_biliary_disease", "steatohepatitis", "chronic_venous_outflow_obstruction", "autoimmune_hepatitis", "a1at_deficiency", "cirrhosis", "primary_biliary_cholangitis", "acute_hepatitis", "paracetamol", "granulomas", "cholestatic_liver_injury", "large_duct_obstruction", "fatty_liver_disease", "congenital_hepatic_fibrosis", "haemochromatosis", "non-cirrhotic portal hypertension", "graft_verusus_host_disease", "chronic_hepatitis_B_virus", "chronic_hepatitis_C_virus", "iron_overload", "primary_sclerosing_cholangitis", "primary_sclerosing_cholangitis_steatosis", "primary_biliary_cholangitis_autoimmune_hepatitis_overlap", "autoimmune_liver_disease", "extramedullary_haematopoiesis", "graft_versus_host_disease", "acute_cholestatic_hepatitis", "non-cirrhotic_portal_hypertension", "complete_response_to_chemo"
